# The Effects of an Online–Offline Hybrid Exercise Program on the Lives of Children with Cerebral Palsy Using Wheelchairs during the COVID-19 Pandemic in Korea

**DOI:** 10.3390/ijerph19127203

**Published:** 2022-06-12

**Authors:** Youngshin Lim, Areum Han, Mingoo Lee, May Kim

**Affiliations:** 1 Department of Physical Education, College of Education, Korea University, Seoul 02841, Korea; enakore@korea.ac.kr; 2 Department of Sport Sciences, College of Energy and Biotechnology, Seoul National University of Science & Technology, Seoul 01811, Korea; aruemee@naver.com; 3 College of Medicine, Korea University, Seoul 02841, Korea; mingoolee@korea.ac.kr

**Keywords:** children with CP, COVID-19, online exercise programs, reintegration to normal living (RNL), social interaction, quality of life

## Abstract

Due to the ongoing COVID-19 pandemic, many online programs for social meetings, education, leisure, and physical activities have been developed and provided; however, children with cerebral palsy (CP) cannot enjoy online programs in the same way that those without disabilities can. The aim of this study was to investigate the differences in reintegration to normal living (RNL), social interaction, and quality of life among school-age children with CP after participation in a game-based online–offline hybrid group exercise program. The current study was conducted on 26 children with CP who participated in a hybrid exercise program. The RNL, social interaction, and quality of life were measured before and after the six-week program. The scores of RNL and quality of life were improved (*p* < 0.05) after program participation. Online or hybrid exercise programs incorporating interactive methods (i.e., competition and cooperating) could enhance RNL and quality of life of children with CP. Thus, well-designed online or hybrid exercise programs should be developed and provided for children with CP to enhance overall quality of life during the pandemic.

## 1. Introduction

The ongoing COVID-19 pandemic has affected people’s lives and led to changes in the daily lives of the worldwide population. People with disabilities who have pre-existing barriers are more vulnerable to unexpected pandemics than people without disabilities. Specifically, children with disabilities and their caregivers have struggled more with changes and adapting to unanticipated pandemic situations than the general population has [1,2]. Social-distancing measures and restrictions on group facilities and gatherings (e.g., school closures and curfew-by-district) were implemented in South Korea by the government to contain the spread of COVID-19 infections [3]. Such protective measures and restrictions have directly affected community-based services that children with disabilities often rely on, for example, health care systems, disability services, institutional environments, and schools [4]. People with disabilities and their families may be disproportionately affected by disrupted access to health care and community support services in these extreme circumstances, resulting in increased stress and anxiety and decreased outdoor and physical activity [5,6]. The suffering and stress might be even higher for children with severe disabilities, such as children with cerebral palsy (CP) or children relying mainly on wheelchairs for their mobility, in pandemic situations because it is almost impossible for them to ambulate outside independently and because they are more likely to experience access limitations [7,8].

In addition, the lack of physical activity and regular rehabilitation treatment due to COVID-19 negatively affect physical development and the attenuation of physical strength, such as muscle loss and muscle atrophy among children with CP, which may cause other problems such as lethargy and social isolation [9]. Children with CP are more likely to have limited opportunities to interact with peers due to their disability and limitations in mobility and speech [10], which have likely worsened during the COVID-19 pandemic. Thus, this study particularly focused on school-aged children with CP who mainly used a manual wheelchair for mobility and who were considered to be more negatively affected by COVID-19 social-distancing measures since they have dependent mobility. Numerous researchers have identified various physical, social, and psychological health benefits that occur among children with CP when participating in physical and leisure activities [11,12,13,14]. Yun and Kim [15] reported that both middle school and high school students with physical disabilities who regularly participated in exercise had higher levels of self-esteem and social adjustment (responsibility, impulsiveness, adaptability, and sociality) than those who did not participate in exercise, and happiness levels were higher. Similarly, Han et al. [16] highlighted the importance of physical leisure activities as a means of enhancing positive feelings and emotions and forming social support that can lead to a higher quality of life among people with physical disabilities. Other benefits of physical activity, such as enhancing motor function, mobility, and social interaction, contributing to quality of life and happiness among children with CP are also well reported [11,14,17].

Physical activity is also a significant predictor of satisfaction with the performance of the daily activities of people with disabilities. The Reintegration to Normal Living (RNL) Index has been used to assess personal satisfaction with social, physical, and psychological functions in the everyday activities of people with disabilities. The RNL is particularly important to understanding the daily life of people with disabilities since it shows how one can actively participate in some domains of human life, such as mobility, self-care, daily activity, and recreational activity [18]. McVeigh et al. [19] revealed that sports participants with spinal cord injuries reported higher community integration and RNL than non-sports participants with spinal cord injuries. Additionally, Crawford et al. [20] found that people with mobility impairment who engaged in a high level of physical activity reported higher participation in major life events, better health, and a higher level of RNL than did mobile-impaired people with low or non-existing physical activity levels. Specifically for those using a wheelchair, exercises for upper limb strength could enhance their strength to control the wheelchair, which might contribute to the increase of independent mobility [21] and the activities of daily living (ADL) [22] and eventually improve social participation, RNL, and quality of life [20].

Despite the known benefits of participation in physical activities, the physical activity levels of people with or without disabilities have dropped due to the COVID-19 pandemic [6,23]. As alternatives under the COVID-19 quarantine system, the digital accessibility and utilization of social-media-based social connections, home training, and online programs are increasing [24,25]. However, the method and system of digital communication may not be accessible without assistants, which is a considerable limitation to online contact, particularly for people with severe disabilities [26,27]. Even if online fitness programs are accessible, internet-based physical activity programs have not been very effective for children with CP [28]. Additionally, home-based training showed no or limited effects on the social participation in recreation and leisure, self-perception, and quality of life of children with CP [22]. Thus, it is necessary to understand the characteristics of children with CP and develop a customized online exercise program that facilitates children’s enjoyment and ongoing participation [29]. Although the focus of the program was more on online program delivery, the majority of children with severe disabilities were not accustomed to the online exercise program with other participants; thus, we added a couple of offline sessions. After considering existing literature, COVID-19 restrictions, and the characteristics of the participants, we applied a game-based online–offline group exercise program focusing on upper-body strength exercises for children with CP using a wheelchair. The upper-body strength exercise was chosen because it was the most necessary and beneficial exercise for wheelchair users [21] and could increase the quality of life [30]. In addition, many physical education or physical activity-related literature has shown that game-based group physical activity programs have been effective to increase teamwork and socialization [31,32]; thus, it is necessary to explore if the same effects exist for an online-focused program, especially for children with CP. During the program, the children were intended to experience teamwork while competing with other teams. We were particularly interested in whether the program would positively influence children’s RNL, social interaction levels, and quality of life.

Therefore, the purpose of this study was to explore the effectiveness of the program and investigate the differences in RNL, social interaction levels, and quality of life among school-age children with CP after participation in a game-based online–offline hybrid group exercise program. The hypotheses were as follows.

**Hypothesis** **1.** *There will be differences in RNL among children with CP before and after participation in a blended (online and offline) group exercise program*.

**Hypothesis** **2.** *There will be differences in social interaction levels among children with CP before and after participation in the blended group exercise program*.

**Hypothesis** **3.** *There will be differences in the quality of life of children with CP before and after participation in the blended group exercise program*.

## 2. Materials and Methods

The pre–post research design was employed to observe the association between a six- week game-based online–offline hybrid group exercise program for children with CP to explore the effects of the program on children’s RNL, social interaction levels, and quality of life.

### 2.1. Participants

Participants were recruited from children with CP who were provided wheelchairs from a mobility-improvement project of a welfare foundation. Among them, only children whose parents or caregivers could support online–offline exercise participation were able to participate in this program due to its requirement of both online and offline participation. Thirty-three children voluntarily participated in a group exercise program for CP organized by a G fitness center for children with disabilities in Seoul, South Korea, but only 26 children participated in all six-week sessions and completed both pre- and post-program surveys. Participants were allowed to continue any rehabilitation treatments that they had undergone before participating the program.

Throughout the program, there were unpredictable changing schedules due to the government’s COVID-19 policy, making it difficult for the children’s parents and caregivers to follow the changed schedules to support their child’s participation. Additionally, some parents experienced challenges in providing support for their child’s online participation due to a lack of technical knowledge, which also caused an increase in the dropout rate. Moreover, children with disabilities or chronic conditions, including children with CP, are often considered vulnerable during such pandemics by their parents or caregivers [7,33]; thus, it was very difficult to recruit participants for any studies requiring social contacts or visiting unfamiliar places. Accordingly, the sample size of the study seems relatively small; however, it still accounted for 0.52% of the total population of school-aged children with brain lesions in South Korea [34].

Participants’ ages ranged from 6 to 15 years (M = 11.1, SD 2.29), with 57.7% identifying as male. The degree of brain lesion disability was reported by parents or caregivers based on the Korean disability rating system, which was assessed by the Modified Barthel Index (see Kim et al., 2018 [35]). The participants ranged from level II (a person who needs the help of others in most of their daily lives and walking, Modified Barthel Index score range 33–53) to level IV (a person who can perform most necessary daily movements but needs help from others intermittently, Modified Barthel Index score range 70–80), which is close to GMFCS levels 4–5. Participation in the study was voluntary with the option to withdraw at any time.

### 2.2. Procedure

The purpose of the study was explained to the parents and caregivers, and informed consent was obtained from them. Parents/caregivers completed questionnaires assessing children’s RNL, social interaction levels, and quality of life before starting the program. Parents/caregivers of children with CP who participated in the exercise program were asked to complete the self-administered questionnaires via email or Google online survey.

The game-based online–offline hybrid group exercise program was conducted for 6 weeks every Sunday between September 2020 to October 2020. The program consisted of four online sessions (1st week, 2nd week, 3rd week, and 6th week) and two offline sessions (4th week and 5th week), which lasted for 50 min each. The online sessions were conducted using video conferencing platforms, and the offline sessions were held at a gym in Seoul, South Korea. Participants were divided into five classes (5 to 7 children) which eventually became five teams for competition prior to beginning the program. The exercise program focused on a different set of physical activities using metal cage-shaped exercise equipment allowing height and weight adjustments to fix the resistance bands according to the physical characteristics of individual users. The equipment was developed by a team of medical doctors with national funding to support the exercise participation of people with disabilities and was used for exercise programs by wheelchair users [36]. Each group exercise session was led by an instructor and a support person. Both of them were physiotherapists and exercise experts who had experience working with children with CP. When the online sessions were held, caregivers’ support was needed at their home.

Each online session included (1) a warm-up exercise (stretching), (2) five movements of muscle-strengthening exercises focused on the upper body using resistance bands fixed on a metal cage-shaped exercise instrument, and (3) a cool-down exercise. Strengthening exercises included shoulder-press, pull-down, and trunk exercises [36] that were developed to enhance upper body strength for wheelchair users (e.g., Van Straaten, 2014 [37]). The offline sessions were conducted for group (team) competition. Each participant’s score (the number of the five movements) was added to a team score, and the team scores were compared in a team sports match to encourage active participation and motivate participants (e.g., Zhang et al., 2016 [38]). In order to encourage exercise participation and enhance teamwork, the instructor continuously reminded the participants in each class that they belonged to the same team for the offline competition and that their participation levels would matter to win the competition. Additionally, the video instructing the five exercise movements was provided to participants for home training to encourage exercise for the team competition. The level of home training participation was asked; however, it was not considered for the study because the focus of the study was to explore not the physical condition but the social aspects of the participants. No injuries or special concerns caused by exercise occurred during the online or offline exercise sessions.

### 2.3. Measurement Instruments

The participants’ level of community reintegration was measured with the Reintegration to Normal Living Index (RNLI), which consists of 11 items in eight domains [39]. The RNLI assesses global function and measures both the patients’ perceptions of their own capabilities and the objective indicators of physical, social, and psychological performance. The RNLI was initially developed to assess the satisfaction level of performing daily activities by those who complete rehabilitation programs. However, it has also been used in people with mobility impairments, including cerebral palsy and poliomyelitis, and has been shown to have good reliability and validity [18]. Two items on the scale were slightly modified based on the children’s characteristics and daily living contexts. The items are scored from 1 point (strongly disagree) to 5 points (strongly agree). Higher scores indicated higher participation in daily living. The RNLI has a high internal consistency (Cronbach’s alpha = 0.722).

Although RNLI includes an item indirectly asking the sociality of the respondents, we used four additional items to identify changes in participants’ ability to interact with various social contexts. The items measuring the social interaction levels of the participants were modified from the socialization part of the Children’s Assessment of Participation and Enjoyment (CAPE) [40] used in many studies for children with CP. The CAPE was developed to measure the different dimensions of the participation level (i.e., numbers of activities, frequency of practicing, social contexts, physical contexts, and enjoyment); however, we only utilized the social context dimension because the other dimensions were identical for all participants. The original social context dimension was measured by scoring 1–5 in different social contexts, including “alone”, “with family”, “with relatives”, “with friends”, and “with others”. However, we modified the dimension to 4 levels, “with family members” (1 point), “with friends” (2 points), “with teachers/instructors” (3 points), and “with others” (4 points), and the social context was limited to conversation, with each context based on the characteristics and situations of the participants. The item of each level was asked using a binary nominal scale of yes or no answers; then, the points of each level were added to calculate the total scores. Higher scores indicated higher social interaction levels.

For assessing the quality of life, the work of Charlifue et al. [41] for people with spinal cord injuries was translated and used. Items for evaluating the quality of life were rated on a scale of 10 points (1 point: strongly disagree, to 10 points: strongly agree), with three questions about overall life satisfaction, satisfaction with physical health, and satisfaction with psychological health, emotions, and mood. The higher the measured score was, the higher the quality of life was (Cronbach’s alpha = 0.913). The original scales were developed in English; thus, the back translation method was utilized. Although the measurements used in this study have rarely been used among children and were not validated in Korean, most items were comprised of everyday words; thus, no problems occurred in administrating the survey. Additionally, all the items were modified in a form that parents/caregivers could respond to, and parents/caregivers were asked to assess their children’s statuses. All of the scales used and the final version of the survey were reviewed by expert panels for content validity.

The pre- and post-survey questionnaire for this study consisted of a total of 18 questions (i.e., 11 questions about RNL, 4 about social interaction level, and 3 about quality of life) and general characteristic questions. Demographic information (i.e., gender, age, and disability grade) was also asked in the pre-survey (Table 1).

### 2.4. Data Analysis

The data collected in this study were analyzed using IBM SPSS Statistics for Windows, version 25.0 (IBM Corp., Armonk, NY, USA) Frequency analysis and technical statistics were conducted to identify demographic information, participation in exercise programs, RNL, social interaction levels, and quality of life of the participants. Before statistical analysis, the Shapiro–Wilk test was performed to assess the normality of continuous data. The results did not show evidence of non-normality of RNL (*W*(26) = 0.982, *p* = 0.913) and quality of life (*W*(26) = 0.970, *p* = 0.620). However, the social interaction levels did not meet the criteria for the normality assumption; thus, the Wilcoxon signed-rank test was used to identify the differences between pre- and post-program scores. A paired t-test analysis comparing pre- and post-program scores was conducted to identify changes in the RNL and quality of life of children with CP before and after participating in the exercise program. We adopted a statistical significance level at *p* *≤* 0.05. The differences between pre- and post-program tests were presented using means, standard deviations, t-values for the paired t-test, z-values for the Wilcoxon signed-rank test, and *p*-values.

## 3. Results

To explore the differences in RNL, social interaction levels, and quality of life among school-age children with CP after participation in a game-based online–offline hybrid group exercise program, the pre- and post-program tests were conducted. A paired t-test was conducted to determine the difference in RNL of children with CP before and after participating in the exercise program. The average of the pre-program test was 3.20 (*SD* = 0.46), and that of the post-test was 3.40 (*SD* = 0.51), which were significantly different (*t*(25) = −2.210, *p* < 0.05). The average values were compared to see the differences in the detailed items of the RNLI before and after participation in exercise programs for children with CP. In detail, only one item, the ability to deal with life events, showed a significant positive difference before and after participating exercise program (*t*(25) = 2.379, *p* < 0.05). However, the scores of nine RNLI items improved after participating in the program, while those of two items remained same (Table 2). The abilities of dealing with life events, participating in leisure activities, and moving around in the neighborhood improved the most.

A Wilcoxon signed-rank test was conducted to determine the differences in social interaction levels of children with CP before and after participating in the exercise program. The results of the pre-test (*Md* = 10, *n* = 26) and the post-test (*Md* = 10, *n* = 26) were not significantly different, *z* = −1.051, *p* = 0.293 (Table 3). However, participants reported positive changes in the levels of interaction with social contexts although the changes were minimal.

The paired t-test was conducted to determine the difference in the quality of life before and after participation in exercise programs for children with CP. The mean of the pre-test, 6.20 (*SD* = 1.92), and the mean of the post-test, 7.50 (*SD* = 1.39), were significantly different (*t*(25) = −3.765, *p* < 0.01; Table 4). All items showed significant differences after participating in the exercise program, with details as follows: overall satisfaction with life (*t*(25) = −3434, *p* < 0.01), satisfaction with physical health (*t*(25) = −2.929, *p* ≤ 0.01), and satisfaction with psychological health, emotions and mood (*t*(25) = −3.353, *p* < 0.01).

## 4. Discussion and Conclusions

This study aimed to explore the effects of participation in a game-based online–offline hybrid group exercise program designed for children with CP during the COVID-19 pandemic. Their RNL, social interaction levels, and quality of life were compared before and after the six-week exercise program.

The results showed a significant difference in RNL before and after the group exercise program among children with CP. At the item level, the scores of most items improved after program participation, although significant differences among items were only found in dealing with life events. The results of the current study are in line with previous research regarding the effects of exercise participation on the RNL, sociality, and life quality of people with disabilities [42]. Additionally, McVeigh et al. [19] found that people with SCI with higher levels of sports participation were more likely to have increased community integration and quality of life, and Crawford et al. [20] showed that people with mobility impairments who reported high levels of physical activity reported higher RNL and better health. Although the improvement in RNL observed in this study was not dramatic, the finding still suggested that participation in online–offline hybrid exercise programs would benefit children with CP and could generate positive outcomes as offline exercise programs do.

Previous studies showed that group exercise/team sports participation could increase the levels of sociality and socialization for children with CP [11,43]. However, our study did not show positive improvement related to socialization in either RNL or social interaction levels. These different results might be due to many possible reasons. Although the two offline sessions based on a team game format were provided, the opportunities for social interaction were very limited. Since most of the participants were not familiar with the online environment as well as the exercise routine, it was impossible for them to socialize with co-participants during the online session. Further, it was likely that the children participating in the program did not consider the co-participants of the program as friends because they had not known the co-participants before and had met them offline only very limitedly during the program period. If the program was provided for a longer period and the participants had more opportunities to socialize with others during exercise, the positive changes might have increased. In addition, the social interaction levels were asked with a binary nominal scale instead of asking the degree of interaction, which might have influenced the study results as well. Therefore, future research should be undertaken to develop an online or online–offline hybrid group exercise program with an appropriate program design that facilitates social interaction, and research is needed to investigate using a well-known valid measurement method.

Unlike the social interaction level, the children’s quality of life was positively changed after participation in the program. Notably, findings showed significant differences in all aspects of quality of life: overall life satisfaction, satisfaction with physical health, and satisfaction with psychological health, emotions, and mood. These results are in line with numerous studies that reported the effect of offline exercise programs [36,44,45]. Therefore, this study suggests that participation in the game-based online–offline hybrid group exercise contributed to the increased quality of life of children with CP, similarly to previous studies that reported benefits of the participation in the offline group exercise program [36,46]. Children with CP using wheelchairs experience an extreme hardship to visit a gym or to participate in exercise programs specially designed for them [47,48]. Thus, the results showing the effectiveness of program participation on the quality of life are positive signs for online exercise program developers and adaptive sport practitioners, as well as children with CP and their caregivers. Although the program provided in our study might not be an optimal form of replacing an offline team sports program due to many limitations (e.g., limited social interactions), an online or hybrid exercise program could be an alternative for children with CP to enjoy similar positive effects of sports participation. Modified and improved online or hybrid exercise programs for children with CP should be continuously developed and promoted.

There are several limitations. The study had a relatively small sample size and no control group; thus, the results do not provide sufficient evidence for the effectiveness of the exercise program on daily living in social context and quality of life. Due to COVID-19, it was difficult to recruit children with CP who might be more vulnerable to such pandemics than general population. Although the current study results could be meaningful, the interpretation of the results should be done cautiously. Thus, it is necessary to verify the effectiveness of the online or hybrid exercise programs with a sophisticated research design with a large sample. Additionally, unpredictable schedule changes caused by the government’s social-distancing measures were burdensome for participants’ parents or caregivers in supporting their children’s participation in the program. In addition, although active parental involvement could play a significant role in intervention programs for health behaviors [49], the parents’ and caregivers’ lack of technical knowledge made it difficult to support the participants’ online participation, which possibly led to relatively high dropout rates. Many studies on digital health services for people with disabilities or chronic illness actually showed that digital literacy and related support were essential factors for the effectiveness of the program [50]. Therefore, the guidelines or support of online program usage for parents and caregivers should be given in more detail and easier formats in future studies than the ones that were applied in our study.

Another possible limitation of this study is measurement. There were difficulties in finding suitable instruments for the study purpose and subjects, which should be short and easy to answer but could also measure the daily living in social contexts and quality of lives of children with CP. Although we tried to adopt appropriate instruments for these situations, widely used scales with high validity should be investigated or developed and applied to future studies of children with CP. Additionally, the survey was conducted by caregivers or parents of the study participants because it was hard for most of our participants to understand some contents of the survey and answer on the online survey form. Although we instructed the caregivers or parents to answer each item after consulting with their child, some of them might have answered based on their judgement, which might be influenced by the expectation that their children’s states would get better after participating in a new program.

Further, the design of the exercise program in this study was a hybrid form that included some offline sessions; thus, additional studies should be conducted. In addition, the exercise program provided in our study included competition and cooperation as with a team sport, which might have led to the positive effects of program participation. As such, practitioners should consider online exercise programs using a game- or sports-like format for children with CP to guarantee that they receive the positive effects of participation. Recently, telehealth services (using digital information and communication technologies for the health of people with disabilities or chronic condition) have been emerging; however, the research shows conflicting results of the satisfaction and effectiveness of the services [51,52]. Thus, it might be necessary to focus on the quality of the program contents as well as the technical issues of the program. Adopting physical education or adaptive sport contents might be a tip for developing effective and enjoyable online exercise programs for children with CP.

In conclusion, this study focused on exploring the positive changes in participation related to daily living in the social context and quality of life through the hybrid exercise program rather than the enhancement of physical functions of children with CP, which seems small but meaningful; the results of the study could open doors for future research. More studies should be conducted to enhance the quality of online or digital-related exercise programs for children with disabilities and to explore the statistical effectiveness of the programs, which will eventually enable children with disabilities to enjoy the benefits of exercise.

## Figures and Tables

**Table 1:** Pre- and post-survey questions.

Questions	Items
Demographic characteristics	gender, age, and disability grade
Reintegration to normal living (RNLI)	moving around in the house, moving around outside in the neighborhood, being able to take trips out of town, being comfortable with doing what they think is necessary, spending important time in school, being able to participate in recreational activities, being able to participate in social activities, doing what they need at home according to their needs, being comfortable with caregivers, being comfortable with others, and dealing with life events as they happen
Social interaction level	conversations with family members, friends, teachers, and others
Quality of life	overall life satisfaction, satisfaction with physical health, satisfaction with psychological health, emotions, and mood

**Table 2:** Comparison of the RNL between before and after exercise program participation.

Variable	Items	*M*(*SD*)		Change Score	*t*	*p*
		Pre	Post			
Reintegration to Normal Living Index (RNLI)		3.20 (0.46)	3.40 (0.51)	+0.20	−2.210 *	0.036
	moving around in the house	3.81 (0.80)	4.08 (0.69)	+0.27	−1.570	0.129
	moving around outside in neighborhood	2.12 (1.03)	2.46 (1.10)	+0.34	−1.516	0.142
	being able to take trips out of town	4.12 (0.71)	4.19 (0.80)	+0.07	−0.493	0.627
	being comfortable with doing what they think is necessary	2.54 (0.81)	2.54 (0.81)	+0.00	0.000	1.000
	spending the important time in school	2.92 (0.89)	3.00 (0.85)	+0.08	−0.440	0.664
	being able to participate in recreational activities	3.38 (0.90)	3.73 (0.96)	+0.35	−1.735	0.095
	being able to participate in social activities	3.38 (0.87)	3.50 (0.76)	+0.12	−0.618	0.542
	doing what they need at home according to their needs	3.08 (0.85)	3.31 (0.97)	+0.23	−1.140	0.265
	being comfortable with caregivers	4.08 (0.85)	4.31 (0.74)	+0.23	−1.140	0.265
	being comfortable with others	3.15 (0.88)	3.15 (0.93)	+0.00	0.000	1.000
	dealing with life events as they happen	2.62 (0.98)	3.08 (0.85)	+0.46	−2.379 *	0.025

Note: * *p* < 0.05.

**Table 3:** Comparison of social interaction levels between before and after exercise program participation.

Variable	Items	Median		Change Score	*Z*	*p*	n(%)	
		Pre	Post				Pre	Post
Social interaction levels		8.27 (3.16)	8.69 (2.57)	+0.42	−1.051	0.293		
	conversation with family members						25 (96.2%)	26 (100%)
	conversation with friends						22 (84.6%)	22 (84.6%)
	conversation with teachers/instructors						22 (84.6%)	24 (92.3%)
	conversation with others						20 (76.9%)	21 (80.8%)

**Table 4:** Comparison of quality of life between before and after exercise program participation.

Variable	Items	*M* (*SD*)		Change Score	*t*	*p*
		Pre	Post			
Quality of life		6.20 (1.92)	7.50 (1.39)	+1.30	−3.765 **	0.001
	overall life satisfaction	6.35 (2.04)	7.69 (1.29)	+1.34	−3.434 **	0.002
	satisfaction with physical health	5.65 (2.13)	6.96 (1.78)	+1.31	−2.929 **	0.007
	satisfaction with psychological health, emotions, and mood	6.58 (2.06)	7.85 (1.60)	+1.27	−3.353 **	0.003

Note: ** *p* < 0.01.

## Data Availability

The data presented in this study are available from the corresponding author upon reasonable request. The data are not publicly available due to ethical and privacy restrictions.

## References

[1] Bentenuto A., Mazzoni N., Giannotti M., Venuti P., de Falco S. (2021). Psychological impact of COVID-19 pandemic in Italian families of children with neurodevelopmental disorders. Res. Dev. Disabil.

[2] Paulauskaite L., Farris O., Spencer H.M., Hassiotis A., EPICC-ID group (2021). My Son Can’t Socially Distance or Wear a Mask: How Families of Preschool Children with Severe Developmental Delays and Challenging Behavior Experienced the COVID-19 Pandemic. J. Ment. Health Res. Intellect. Disabil..

[3] Korea Ministry of Health and Welfare Overview of Social Distancing System. http://ncov.mohw.go.kr/en/socdisBoardView.do?brdId=19&brdGubun=191&dataGubun=191&ncvContSeq=&contSeq=&board_id=.

[4] World Health Organization (2020). Disability Considerations during the COVID-19 Outbreak.

[5] Lebrasseur A., Fortin-Bédard N., Lettre J., Bussières E.-L., Best K., Boucher N., Hotton M., Beaulieu-Bonneau S., Mercier C., Lamontagne M.-E. (2021). Impact of COVID-19 on people with physical disabilities: A rapid review. Disabil. Health J..

[6] Sutter E.N., Francis L.S., Francis S.M., Lench D.H., Nemanich S.T., Krach L.E., Sukal-Moulton T., Gillick B.T. (2021). Disrupted Access to Therapies and Impact on Well-Being during the COVID-19 Pandemic for Children With Motor Impairment and Their Caregivers. Am. J. Phys. Med. Rehabil..

[7] Cankurtaran D., Tezel N., Yildiz S.Y., Celik G., Akyuz E.U. (2021). Evaluation of the effects of the COVID-19 pandemic on children with cerebral palsy, caregivers’ quality of life, and caregivers’ fear of COVID-19 with telemedicine. Ir. J. Med. Sci..

[8] Rodby-Bousquet E., Hägglund G. (2010). Use of manual and powered wheelchair in children with cerebral palsy: A cross-sectional study. BMC Pediatr..

[9] Lee S.-H., Lee B.-H. (2020). Current Status of Support for the Disabled and Policy Tasks Following the COVID-19 Outbreak. Health Welf..

[10] Stewart M., Barnfather A., Magill-Evans J., Ray L., Letourneau N. (2011). Brief report: An online support intervention: Perceptions of adolescents with physical disabilities. J. Adolesc..

[11] Clutterbuck G.L., Auld M.L., Johnston L.M. (2020). SPORTS STARS: A practitioner-led, peer-group sports intervention for ambulant children with cerebral palsy. Activity and participation outcomes of a randomised controlled trial. Disabil. Rehabil..

[12] Lai B., Lee E., Kim Y., Matthews C., Swanson-Kimani E., Davis D., Vogtle L., Rimmer J.H. (2021). Leisure-time physical activity interventions for children and adults with cerebral palsy: A scoping review. Dev. Med. Child Neurol..

[13] Lauruschkus K., Westbom L., Hallström I.K., Wagner P., Nordmark E. (2013). Physical activity in a total population of children and adolescents with cerebral palsy. Res. Dev. Disabil..

[14] Maher C.A., Toohey M., Ferguson M. (2016). Physical activity predicts quality of life and happiness in children and adolescents with cerebral palsy. Disabil. Rehabil..

[15] Yun S., Kim K.J. (2020). A comparative study of self-esteem, social adjustment, and happiness according to exercise participation of adolescents with physical disabilities. Korean J. Adapt. Phys. Act..

[16] Han A., Kim J., Kim J. (2019). Coping Strategies, Social Support, Leisure Activities, and Physical Disabilities. Am. J. Health Behav..

[17] Clutterbuck G., Auld M., Johnston L. (2019). Active exercise interventions improve gross motor function of ambulant/semi-ambulant children with cerebral palsy: A systematic review. Disabil. Rehabil..

[18] Stark S.L., Edwards D.F., Hollingsworth H., Gray D.B. (2005). Validation of the Reintegration to Normal Living Indexin a population of community-dwelling people with mobility limitations. Arch. Phys. Med. Rehabil..

[19] McVeigh S.A., Ma S.L.H., Craven B.C. (2009). Influence of sport participation on community integration and quality of life: A comparison between sport participants and non-sport participants with spinal cord injury. J. Spinal Cord Med..

[20] Crawford A., Hollingsworth H.H., Morgan K., Gray D.B. (2008). People with mobility impairments: Physical activity and quality of participation. Disabil. Health J..

[21] Zwinkels M., Verschuren O., Janssen T.W., Ketelaar M., Takken T., Sport-2-Stay-Fit study group (2014). Exercise training programs to improve hand rim wheelchair propulsion capacity: A systematic review. Clin. Rehabil..

[22] Rameckers E.A.A., Janssen-Potten Y.J.M., Essers I.M.M., Smeets R.J.E.M. (2015). Efficacy of upper limb strengthening in children with Cerebral Palsy: A critical review. Res. Dev. Disabil..

[23] Lai B., Wen H., Sinha T., Davis D., Swanson-Kimani E., Wozow C., Young R., Powell D., Rimmer J.H. (2022). The impact of COVID-19 on the lifestyles of adolescents with cerebral palsy in the Southeast United States. Disabil. Health J..

[24] Füzéki E., Schröder J., Reer R., Groneberg D.A., Banzer W. (2022). Going Online?—Can Online Exercise Classes during COVID-19-Related Lockdowns Replace in-Person Offers?. Int. J. Environ. Res. Public Health.

[25] Moreira-Neto A., Martins B., Miliatto A., Nucci M.P., Silva-Batista C. (2021). Can remotely supervised exercise positively affect self-reported depressive symptoms and physical activity levels during social distancing?. Psychiatry Res..

[26] Kim J., Jung G., Han H., Choi H. (2020). Exploring Disability Inclusive Community Resilience Post-COVID-19 Learning from Experiences of Caregivers in Daegu Region. Korean J. Clin. Psychol..

[27] Park J.W. (2020). The Reality and Problems of Non-face-to-face Instruction according to the COVID-19 Situation from The Perspective of College Students with Disabilities. Spec. Educ. Res..

[28] Maher C.A., Williams M.T., Olds T., Lane A.E. (2010). An internet-based physical activity intervention for adolescents with cerebral palsy: A randomized controlled trial. Dev. Med. Child Neurol..

[29] Lai C.-J., Liu W.-Y., Yang T.-F., Chen C.-L., Wu C.-Y., Chan R.-C. (2015). Pediatric Aquatic Therapy on Motor Function and Enjoyment in Children Diagnosed With Cerebral Palsy of Various Motor Severities. J. Child. Neurol..

[30] Chow J.W., Levy C.E. (2011). Wheelchair propulsion biomechanics and wheelers’ quality of life: An exploratory review. Disabil. Rehabil. Assist. Technol..

[31] Anderson D. (2009). Adolescent Girls’ Involvement in Disability Sport: Implications for Identity Development. J. Sport Soc. Issues.

[32] Koekoek J., Knoppers A. (2015). The role of perceptions of friendships and peers in learning skills in physical education. Phys. Educ. Sport Pedagog..

[33] Aishworiya R., Kang Y.Q. (2021). Including Children with Developmental Disabilities in the Equation During this COVID-19 Pandemic. J. Autism Dev. Disord..

[34] Korean Statistical Information Service. https://kosis.kr/index/index.do.

[35] Kim S.W., Jeon H.R., Kim Y., Choi S.J., Youk T., Kim J. (2018). Disability Registration State of Children With Cerebral Palsy in Korea. Ann. Rehabil. Med.-Arm..

[36] Kim S.-H., Shin H.-J., Hahm S.-C., Park S.-W., Cho H.-Y., Lee M.-G. (2020). The Effect of a Program Combining Resistance Exercise and Group Exercise on Balance, Grip Strength, and Quality of Life of Children with Cerebral Palsy. J. Korean Soc. Phys. Med..

[37] Van Straaten M.G., Cloud B.A., Morrow M.M., Ludewig P.M., Zhao K.D. (2014). Effectiveness of Home Exercise on Pain, Function, and Strength of Manual Wheelchair Users With Spinal Cord Injury: A High-Dose Shoulder Program With Telerehabilitation. Arch. Phys. Med. Rehabil..

[38] Zhang J., Brackbill D., Yang S., Becker J., Herbert N., Centola D. (2016). Support or competition? How online social networks increase physical activity: A randomized controlled trial. Prev. Med. Rep..

[39] Wood-Dauphinee S.L., Opzoomer M.A., Williams J.I., Marchand B., Spitzer W.O. (1988). Assessment of global function: The Reintegration to Normal Living Index. Arch. Phys. Med. Rehabil..

[40] King G.A., Law M., King S., Hurley P., Hanna S., Kertoy M., Rosenbaum P. (2007). Measuring children’s participation in recreation and leisure activities: Construct validation of the CAPE and PAC. Child. Care Health Dev..

[41] Charlifue S., Post M., Biering-Sørensen F., Catz A., Dijkers M., Geyh S., Horsewell J., Noonan V., Noreau L., Tate D. (2012). International Spinal Cord Injury Quality of Life Basic Data Set. Spinal Cord.

[42] Diaz R., Miller E.K., Kraus E., Fredericson M. (2019). Impact of Adaptive Sports Participation on Quality of Life. Sports Med. Arthrosc. Rev..

[43] Lee E.J., Song J.M. (2010). Changes in the Gross Motor Function, Self-esteem and Social Ability of Children with Spastic diplegia from Group Exercise: Case Study. J. Korean Soc. Phys. Med..

[44] Okoye E.C., Onwuakagba I.U., Ani K.U., Babatunde J.K., Akosile C.O., Okeke M.C., Aronu A.E. (2020). Body image, physical activity, quality of life, and community reintegration of individuals with acquired mobility disability in a Nigerian population. Disabil. Rehabil..

[45] Shikako-Thomas K., Dahan-Oliel N., Shevell M., Law M., Birnbaum R., Rosenbaum P., Poulin C., Majnemer A. (2012). Play and be happy? Leisure participation and quality of life in school-aged children with cerebral palsy. Int. J. Pediatr..

[46] Verschuren O., Ketelaar M., Gorter J.W., Helders P.J.M., Uiterwaal C.S.P.M., Takken T. (2007). Exercise training program in children and adolescents with cerebral palsy. Arch. Pediatrics Adolesc. Med..

[47] Steinhardt F., Ullenhag A., Jahnsen R., Dolva A.S. (2021). Perceived facilitators and barriers for participation in leisure activities in children with disabilities: Perspectives of children, parents and professionals. Scand. J. Occup. Ther..

[48] Shimmell L.J., Gorter J.W., Jackson D., Wright M., Galuppi B. (2013). “It’s the Participation that Motivates Him”: Physical Activity Experiences of Youth with Cerebral Palsy and Their Parents. Phys. Occup. Ther. Pediatrics.

[49] Rose T., Barker M., Jacob C.M., Morrison L., Lawrence W., Strömmer S., Vogel C., Woods-Townsend K., Farrell D., Inskip H. (2017). A Systematic Review of Digital Interventions for Improving the Diet and Physical Activity Behaviors of Adolescents. J. Adolesc. Health.

[50] Falter M., Scherrenberg M., Kindermans H., Kizilkilic S., Kaihara T., Dendale P. (2022). Willingness to participate in cardiac telerehabilitation: Results from semi-structured interviews. Eur. Heart J. Digit. Health.

[51] Camden C., Pratte G., Fallon F., Couture M., Berbari J., Tousignant M. (2020). Diversity of practices in telerehabilitation for children with disabilities and effective intervention characteristics: Results from a systematic review. Disabil. Rehabil..

[52] Dias J.F., Oliveira V.C., Borges P.R.T., Dutra F., Mancini M.C., Kirkwood R.N., Resende R.A., Sampaio R.F. (2021). Effectiveness of exercises by telerehabilitation on pain, physical function and quality of life in people with physical disabilities: A systematic review of randomised controlled trials with GRADE recommendations. Br. J. Sports Med..

